# Clinical impact of circulating miR-133, miR-1291 and miR-663b in plasma of patients with acute myocardial infarction

**DOI:** 10.1186/1746-1596-9-89

**Published:** 2014-05-01

**Authors:** Liu Peng, Qiu Chun-guang, Li Bei-fang, Ding Xue-zhi, Wang Zi-hao, Li Yun-fu, Dang Yan-ping, Liu Yang-gui, Li Wei-guo, Hu Tian-yong, Huang Zhen-wen

**Affiliations:** 1 Department of Cardiology, the First affiliated Hospital of Zhengzhou University, Zhengzhou 450052, China; 2 Department of Cardiology, Hebi City People’s Hospital, Hebi 458000, China; 3 Department of Cardiology, Henan Province Chest Hospital, Zhengzhou 450003, China

**Keywords:** Acute myocardial infarction, Micro RNA, Diagnosis, Biomarker

## Abstract

**Background:**

Acute myocardial infarction (AMI) is one of the leading causes for death in both developed and developing countries and it is the single largest cause of death in the United States, responsible for 1 out of every 6 deaths. The objective of this study was to determine microRNA (miRNA) expression in AMI and determine whether miR-133, miR-1291 and miR-663b could be measured in plasma as a biomarker for recurrence.

**Methods:**

Patients with AMI and those without AMI were retrospectively recruited for a comparison of their plasma miR-133, miR-1291 and miR-663b expression.

**Results:**

miR-133, miR-1291 and miR-663b levels were significantly overexpressed in AMI compared with Non-AMI. MiR-133 showed an AUC of 0.912, with a sensitivity of 81.1% and a specificity of 91.2%. The AUC for miR-1291 was 0.695, with a sensitivity of 78.4% and a specificity of 89.5%. The AUC for miR-663b was 0.611, with a sensitivity of 72.4% and a specificity of 76.5%.

**Conclusions:**

This study demonstrated that the levels of miR-133, miR-1291 and miR-663b are associated with AMI. The potential of these miRNAs as biomarkers to improve patient stratification according to the risk of AMI and as circulating biomarkers for the AMI progonos warrants further study.

**Virtual Slides:**

The virtual slide(s) for this article can be found here: http://www.diagnosticpathology.diagnomx.eu/vs/8183629061241474

## Background

Acute coronary syndrome (ACS) is a very common health problem in the all the world. Acute myocardial infarction (AMI) is one of the leading causes for death in both developed and developing countries and it is the single largest cause of death in the United States, responsible for 1 out of every 6 deaths [1]. Up to 10 million Americans report to an Emergency Department (ED) for chest pain yearly and this number is expected to climb with continued aging of the United States (U.S.) population [2]. The substantial number of emergent hospitalizations for ACS, including ST-elevation myocardial infarction (STEMI), Non ST-elevation myocardial infarction (NSTEMI), and unstable angina (UA), represents a significant financial strain to both patients and the healthcare system [3]. This burden could be ameliorated, and more efficient treatment administered, if a more rapid, sensitive, and specific biomarker could differentiate between the spectrum of ACS (i.e. STEMI, NSTEMI, and UA) as well as differentiate from other less concerning causes of chest pain, such as gastroesophageal reflux (GERD). Rapid and correct diagnosis of AMI plays an important role in therapy and prognosis for this disease [4]. Over the past two decades, huge progress has been made in the diagnosis, treatment, and prognosis of AMI. Particularly, the progress in biomarkers for AMI attracted a great deal of attention. Currently, cardiac troponins are the most common biomarkers used for diagnosis of AMI in clinical practice [5]. However, there is still a clinical need for novel biomarker, which is able to reliably rule in or rule out AMI immediately on admission.

MicroRNAs (miRNAs) are small (19-22-nt) single stranded non-coding RNA molecules that are derived from hairpin-structured precursors [6,7]. These microRNAs function by directly binding to heir potential target site in the 3′ untranslated region (3′ UTRs) of specific target mRNA, leading to the repression of mRNA translation or the degradation of target mRNAs. Small non-coding RNAs were also recently implicated in control of DNA damage response. Currently, there are 24521 mature miRNA sequences of both human and other species listed in the miRNA registry (Sanger miRBase; http://www.mirbase.org/). Over recent years, many miRNAs have been investigated in various human disease, such as kinds of cancer and cardiovascular diseases [7,8]. The deregulation of expression of microRNAs has been shown to contribute to the multistep processes of carcinogenesis in human by modification either of oncogenic or suppressor gene function [9]. Nowadays, microRNA expression patterns are known to characterize the developmental origins of tumors more effectively than mRNA expression signatures and thus can be a useful tool for the diagnosis and prognosis of human disease [9]. It has long been a goal of cardiovascular research to search for non-invasive tools for the diagnosis and management of AMI that can greatly reduce its worldwide health burden [10]. Recently, it has been reported that microRNAs are circulating in serum/plasma and AMI related microRNAs such as miR-1, miR-133a, miR-208b, miR-223, miR-320a, miR-451 and miR-499 have been detected in the plasma or serum of AMI patients [11,12]. These circulating microRNAs can be considered as a new class of effective biomarkers where their abundance profile might reflect physiological and/or pathological conditions. Accordingly, several subsequent studies have proven that miRNAs can serve as potential biomarkers for various cardiovascular diseases, such as AMI.

The purpose of the present study was to characterize the levels of miRNAs including miR-133, miR-1291 and miR-663b in the circulation of AMI patients. Accordingly, an extensive detection of miRNAs was screened for in the AMI patients and the results were compared with an age-, gender-, and ethnically matched control group. We aimed to develop find the diagnostic value of there miRNAs for the AMI.

## Methods

### Ethics statement

The study was approved by First affiliated Hospital of Zhengzhou University. Written informed consent was obtained for the acquisition and use of patient tissue samples and anonymized clinical data.

### Patient and control cohorts

All patients presenting with suspected AMI pain in the coronary care unit at the First affiliated Hospital of Zhengzhou University from Jan 2008 to Jan 2009, were offered to take part in the study. Among 397 AMI cases, 205 cases were included in this study. See Table 1 for patient characteristics. ST-elevation myocardial infarction (STEMI) diagnosis was based on ECG criteria, non-STEMI (NSTEMI) on troponin levels together with clinical symptoms of ischemia. Non-MI was defined as absence of troponin-elevation and absence of ST-elevation. A peripheral venous blood sample was drawn into tubes containing EDTA, plasma was prepared as described above and stored in liquid nitrogen until analyzed. 71% of blood samples were taken within 24 hours, 82% within 48 hours and 93% within 72 -hours. In 90% of the patients, samples were obtained after interventional therapy. 95% of patients were treated with percutaneous coronary intervention (PCI). Left ventricular ejection fraction (LVEF) was assessed with echocardiography before discharge. Samples were taken at admission, at 3, 10 and 20 hours. Daily repeated samples were taken until a peak value had been identified. The disease and personal history were obtained from the patient and previous data. A total of 25 patient diagnoses as STEMI receive an emergency PCI. The post-operative peripheral venous blood sample was drawn into tubes containing EDTA.

**Table 1:** Clinical characters, risk factor and symptoms of the cohort

**Clinicopathological features**	**All cohort (n = 186)**	**AMI cases**		***P***
		**Yes (n = 76)**	**No (n = 110)**	
Age (year)	62.2 (46–88)	64.6 (46–88)	60 (52–81)	0.071
Male	104	43	61	0.282
BMI	27.2 (23–34)	26.9 (24–34)	27.5 (23–32)	0.455
STEMI		25		
NSTEMI		51		
**Disease and personal history**				
Hypertension	122	74	48	0.341
Hypercholesterolemia	104	54	50	**0.033**
Diabetes	88	43	45	0.237
Smoking	104	42	62	0.569
Alcohol drinking	92	36	56	0.382
Previous AMI	34	19	15	**0.020**
Previous PTCA	25	15	10	**0.001**
**Physical examination**				
Systolic blood pressure, mmHg	138 (121–185)	139 (121–179)	141 (127–185)	0.875
Diastolic blood pressure, mmHg	83 (72–103)	83 (75–99)	84 (72–103)	0.531
Heart rate – beats per minute	75 (62–145)	79 (67–145)	73 (62–91)	0.129
**Electrocardiographic findings**				
T-wave inversion	25	21	4	**< 0.001**
ST-segment change	46	36	10	**< 0.001**
Other Changes	18	9	9	**< 0.001**
No significant changes	97	10	87	**< 0.001**

BMI: body mass index; AMI: acute myocardial infarction; STEMI: ST-segment-elevation; NSTEMI: non-ST-segment elevation; PTCA, Percutaneous transluminal coronary angioplasty.

The results with a significant difference were marked in bold.

### Plasma samples

Venous blood was drawn from each patient both on the day of surgery before commencement of the procedure and between 2 and 6 weeks after surgery. Blood was drawn with the Vaccutainer venepuncture system into ethylene diamine tetraacetic acid (EDTA) tubes (Becton, Dickinson and Company, Melbourne, Australia). The first 3 mL were discarded to avoid possible contamination with miRNAs of the skin plug from the puncture. The blood was then centrifuged at 3800 g for 15 minutes. A maximum of 4 hours between the blood draw and separation into plasma was the standardized protocol in this study, although it has been reported that miRNAs are stable for up to 24 hours of incubation at room temperature. The supernatant (plasma) was separated from the cellular layer by pipetting, and the 3 to 5 mm of plasma just above the interphase was sacrificed to prevent disturbance of the cellular layer. The plasma was stored at 280°C until RNA extraction.

### RNA extraction

Total RNA was extracted from 200lL of thawed plasma with the miRNeasy Mini Kit (Qiagen), in accordance with the manufacturer’s instructions. The RNA was eluted with 50 mL of RNAse-free water and was stored at -70°C until RT-PCR reaction. We quantified the concentration of all RNA samples using NanoDrop 1000 (Thermo Scientific, Wilmington, DE, USA).

### Quantitative reverse transcriptase-polymerase chain reaction

The expression levels of individual miRNAs were measured with quantitative reverse transcriptase-polymerase chain reaction (qRT-PCR) using TaqMan miRNA assays (Applied Biosystems, Foster City, Calif). The relative expression (RQ) was obtained using the ΔΔCt method. Briefly, total RNA was reverse transcribed to complementary DNA (cDNA) using TaqMan miRNA primers according to the manufacturer’s instructions. The PCR products were then amplified from cDNA using the TaqMan miRNA probes with the ABI 7900HT Real-time PCR System according to the manufacturer’s instructions. Standard TaqMan miRNA cycling conditions were used, and all samples were run in triplicate. The miR-16 was used as an endogenous control for the plasma samples. All samples across all PCR plates were calibrated against commercially available human thyroid total RNA (Ambion; Life Technologies Corporation, Carlsbad, Calif).

In the present study, expression of the target miRNAs in plasma was normalized relative to the expression of the endogenous control miR-16. Data were analyzed by the ΔCt method: ΔCt = Ct value _(miR-16)_ - Ct value _(miRNA of interest)_. All data were represented by 2^-△Ct^ value.

### Statistical analysis

Differences between groups were assessed by Mann–Whitney U-test, Fisher’s exact test, or chisquare-test, and Steel–Dwass test was performed for multiple comparisons. Paired samples were analyzed by Wilcoxon’s single-rank test. The diagnostic value for differentiating between BTC patients and the control was assessed by calculating the area under the receiver-operator characteristic curve (AUC). Validation of ROC results was performed by the leave-one-out cross-validation method as described before. In the validation, first, by using the subset of all but one sample, we built a ROC model, and defined the cut-off value in such a way that the sum of sensitivity and specificity was maximized. Then, using the cut-off value, the model was used to predict the left-out recorded samples. When this process was repeated for each sample, the prediction was obtained for every record in the dataset using a model that was blind to the predicted observation. All statistical analyses were performed with the use of SPSS 19.0 software (SPSS, Chicago, IL). A P-value of < 0.05 denoted the presence of a statistically significant difference.

## Results

### Patient characteristics

The baseline characteristics of the patients in this study are listed in Table 1. The median age of the all the patients with was 62.2 years (range, 46–88 years) in the whole cohort, 64.6 years (46–88) in AMI group and 60 years (range, 52–81 years) in the non-AMI group. In the AMI group, there were 25 cases with STEMI and 51 cases with NSTEMI.

When the disease and personal history were considered, there were no significant differences in the status of hypertension, diabetes, smoking and alcohol drinking status in the AMI and non-AMI group. However, the rate of hypercholesterolemia was more common in the AMI group (P = 0.033). The history AMI and PTCA was assessed in both groups and the incidence of there two status were more common in the AMI group (P = 0.020 and P = 0.001).

The physical examination showed that no significant differences were observed in the systolic blood pressure, diastolic blood pressure and heart rate between the two groups. However, an advanced study of the electrocardiographic findings showed that the rates of T-wave inversion, ST-segment change and other changes were higher in the AMI group (P < 0.001).

### MiR-133, miR-1291 and miR-663b expression in the cohort

**Figure 1:**
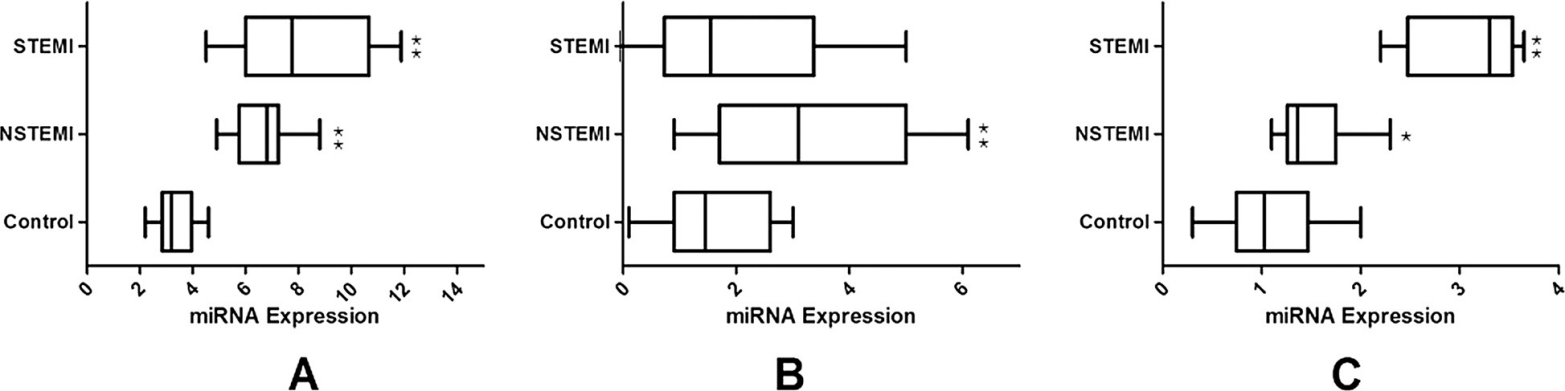
**Levels of the three miRNAs (A: miR-133; B: miR-1291 and C: miR-663b) at presentation in patients with without (n = 931) AMI.** P-values describe the significance level of differences for each miRNA between patients with and without AMI. AMI, acute myocardial infarction; STEMI, ST-segment elevation myocardial infarction; NSTEMI, non-ST-segment elevation myocardial infarction.

The three miRNAs expression was detected in 76 AMI cases (25 STEMI cases and NSTEMI cases) and 110 non-AMI controls normalized to miR-16. As shown in Figure 1, we found that the expression of miR-133 was distinctly increased in STEMI (mean ± SD: 7.6 ± 1.8) and NSTEMI cases (mean ± SD: 7.1 ± 2.8) compared to non-AMI cases (mean ± SD: 3.1 ± 1.3, P < 0.001). In addition, miR-1291 expression in NSTEMI (mean ± SD: 3.3 ± 0.9) was higher than the non-AMI group (mean ± SD: 1.6 ± 1.4, P < 0.01); however, no significant difference was detected in the STEMI group (mean ± SD: 1.8 ± 2.1) compared with the control group (P = 0.06). MiR-663b was distinctly increased in STEMI (mean ± SD: 3.3 ± 1.6) and NSTEMI cases (mean ± SD: 1.4 ± 1.4) compared to non-AMI cases (mean ± SD: 1.0 ± 1.2, P < 0.001).

### Diagnostic performance of miR-133, miR-1291, and miR-663b

**Figure 2:**
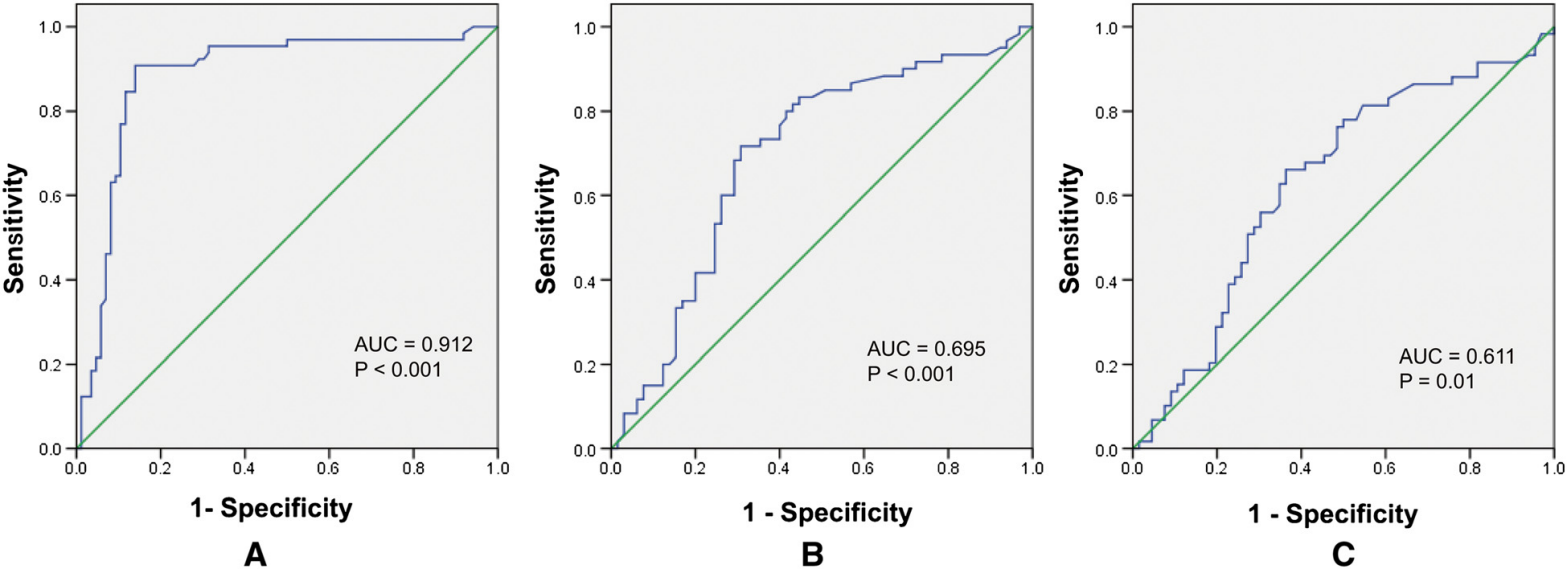
**Receiver operating characteristic curves for the diagnostic accuracy for the three miRNAs. (A)** miR-133, **(B)** miR-1291, **(C)** miR-663b.

The diagnostic performances of miR-133, miR-1291, and miR-663b for the AMI were detected. Cut-off points were determined using the highest sum of sensitivity and specificity. The cut-off points for miR-133, miR-1291, and miR-663b were 4.12, 1.97, and 1.26 respectively. MiR-133 showed an AUC of 0.912, with a sensitivity of 81.1% and a specificity of 91.2% (Figure 2A). The AUC for miR-1291 was 0.695, with a sensitivity of 78.4% and a specificity of 89.5% (Figure 2B). The AUC for miR-663b was 0.611, with a sensitivity of 72.4% and a specificity of 76.5% (Figure 2C).

### The change of the expression after the surgery

**Figure 3:**
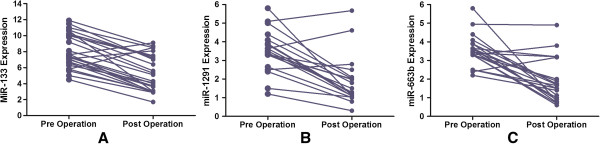
**The change of the expression before and after the surgery. (A)** miR-133, **(B)** miR-1291, **(C)** miR-663b.

A total of 25 STEMI cases were obtained to detect the change of the three miRNAs. Compared with the pre-operative expression, all the three miRNAs demonstrated a significant depression. As showed in Figure 3, the expression of miR-133 changed from 7.6 to 4.3 (P = 0.012). Before the surgery, the expression of miR-1291 was 3.3 ± 0.9 while the post-operative level is 2.1 (P = 0.041). The level of miR-663b changed from 3.3 to 1.2 and a significant difference was detected (P = 0.032).

## Discussion

There is a growing requirement for the detection of a new tool for the AMI. MicroRNAs are small molecules approximately 22 nucleotides in length and have been well conserved evolutionally. Although they are noncoding genes, they are involved in the regulation of a wide range of biologic processes through translational modulation. In cardiovascular pathology, they have been used to classify heart failure and hypertension [13,14]. As well, it was also suggested that miRNA might be a potential biomarker for the diagnosis and prognosis of AMI [12]. In this study, we initially identified the potential biomarker for the diagnosis of AMI. By using qRT-PCR, we confirmed that miR-133, miR-1291 and miR-663b levels were significantly overexpressed in AMI compared with Non-AMI. Although the ROC analyses, we found that there three miRNAs might be potential diagnostic markers for the AMI. Besides, we found that the three miRNAs were significantly decreased post-operatively and which provided a suggestion of the prognosis value for there miRNAs.

MiRNAs have recently been detected in serum or plasma, which are referred to circulating miRNAs [15]. Despite intense research, the origin of circulating miRNAs remains largely unknown. A number of studies have reported that miRNAs are actively secreted in microvesicles or exosomes from different cell types, which area likely source of circulating miRNAs. So far, around 121 miRNAs were identified in exosomes from mast cells, and sphingomyelinase 2 (nSMase2), a rate-limiting enzyme for ceramide biosynthesis, controls the secretion of exosomes to the extracellular matrix [16]. In addition to microvesicles or exosomes, microparticles and lipoprotein complexes (such as high-density lipoprotein complexes) are other possible sources of circulating miRNAs Human miRNAs isolated from plasma are highly stable in boiling water, in solution with very high or low pH [17]. Plasma miRNA levels remain stable when it is subjected to prolonged room temperature incubation or freeze-thawed multiple times. It has been reported that endogenous plasma miRNAs exist in a form that is resistant to plasma Rnase activit. Compared to the endogenous plasma miRNAs, rapid degradation was observed within minutes when synthetic miRNAs (corresponding to caenorhabditis elegans miRNAs cel-miR-39, cel-miR-54, and cel-miR-238) were added into human plasma.

Rapid and correct diagnosis is crucial to treatment and prognosis of AMI. Up to date, cardiac troponins and creatine kinase-MB are the most commonly used biomarkers for AMI, but their clinical value is limited in many cases. Secreted by cardiac cells and accumulated in blood, miRNAs are expected to reflect cardiac injury in response to cardiovascular risk factors and various pathological conditions. Thus, the circulating cardiac-specific or -enriched miRNAs (such as miR-208, miR-499, and miR-133) may provide unique biomarkers for diagnostic and therapeutic interventions of AMI [18]. MiRNA profiling studies in AMI and other diseases have been vastly reported. For instance, a recent tissue miRNA profiling study by Kuwabara Y et al. alrevealed that serum levels of miR-1 and miR-133a were increased significantly in patients not only with acute myocardial infarction but also with unstable angina pectoris and Takotsubo cardiomyopathy without elevation of serum creatine phosphokinase or cardiac troponin [19]. An investigation by By Wang GK, through verifying their tissue expression patterns with real-time PCR analysis, muscle-enriched miRNAs (miR-1, miR-133a, and miR-499) and cardiac-specific miR-208a were selected as candidates for this study. With miRNA microarray and real-time PCR analyses, miR-1, miR-133a, and miR-499 were present with very low abundance, and miR-208a was absent in the plasma from healthy people. In the AMI rats, the plasma levels of these miRNAs were significantly increased [20]. In a microarray study by peripheral total blood samples, identified 121 miRNAs, which are significantly dysregulated in AMI patients in comparison to healthy controls. Among these, miR-1291 and miR-663b show the highest sensitivity and specificity for the discrimination of cases from controls. Using a novel self-learning pattern recognition algorithm, we identified a unique signature of 20 miRNAs that predicts AMI with even higher power (specificity 96%, sensitivity 90%, and accuracy 93%). In addition, we show that miR-30c and miR-145 levels correlate with infarct sizes estimated by Troponin T release [21].

Besides, in this study, we found that the expression of miR-133, miR-1291 and miR-663b were significantly reduced after the surgery. These findings were first reported in this study. The reduction of these miRNAs might indicate a sign of reconditioning of ischemic cardiac muscle. The dynamic expression of these molecular biomarkers may be used as a prognostic marker. Advanced well-designed studies with larger cohort and longer follow-up are required.

## Conclusion

In this study, we identified plasma miR-133, miR-1291 and and miR-663b were significantly increased in the AMI cases. Through ROC analyses, it was found that miR-133, miR-1291 and and miR-663b were potential predictors of AMI. Circulating levels of the same miRNAs also were correlated with the presence of AMI. Advanced well-designed studies are required.

## Competing interests

The authors declare that they have no competing interests.

## Authors’ contributions

QCG and HZW provided the conduction of the whole project, LP, QCG, LBF, DXZ, WZH, LYF, DYP, LYG, LWG, HTY and HZW performed the research, LP, DYP, LYG, LWG, HTY and HZW drafted the manuscript; LP, QCG, LBF, DXZ, WZH, LYF, DYP, LYG, LWG, HTY and HZW contributed to revise the manuscript. All authors read and approved the final manuscript.
